# Trends in the geographic distribution of nursing staff before and after the Great East Japan Earthquake: a longitudinal study

**DOI:** 10.1186/s12960-015-0067-6

**Published:** 2015-08-25

**Authors:** Noriko Morioka, Jun Tomio, Toshikazu Seto, Yasuki Kobayashi

**Affiliations:** Department of Public Health, Graduate School of Medicine, The University of Tokyo, 7-3-1 Hongo, Bunkyo-ku, Tokyo 113-0033 Japan; Center for Spatial Information Science, The University of Tokyo, 4-6-1 Komaba, Meguro-ku, Tokyo 153-8505 Japan

**Keywords:** Great East Japan Earthquake, Nuclear disaster, Geographic distribution, Nurse

## Abstract

**Background:**

Medical care systems in Iwate, Miyagi and Fukushima prefectures were greatly damaged by the Great East Japan Earthquake (GEJE), which struck on 11 March 2011. The shortage of nurses in this area was concerning; however, temporal trends have not been investigated. This study aimed to investigate the trends in the geographic distribution of total nursing staff per population in the secondary medical areas (SMAs) of these prefectures before and after the GEJE. We also aimed to qualify the above trends.

**Methods:**

We conducted a longitudinal study at four time points (July 2007, 2010, 2011 and 2013) over 6 years using reports of basic hospitalization charges from all hospitals within Iwate, Miyagi and Fukushima prefectures that experienced severe damage from the GEJE. We calculated the number of total nursing staff per population in the SMAs and compiled descriptive statistics. Changes from 2010 to 2013 were qualified and mapped.

**Results:**

In coastal SMAs, the ratios of total nursing staff per population decreased immediately after the GEJE. In most SMAs in 2013, the ratios increased and exceeded the pre-GEJE level. However, the changes in total nursing staff per population from 2010 to 2013 were negative in Ryouban (−4.0%), Ishinomaki–Tome–Kesennuma (−1.9%), Sousou (−47.7%) and Iwaki (−1.9%). In Sousou, which is closest to the Fukushima Daiichi Nuclear Power Plant, the changes in total nursing staff per population qualified by job role were −33.7% for nurses, −57.7% for associate nurses and −63.2% for nursing aides.

**Conclusions:**

Our study indicated that the temporal trends in the number of total nursing staff per population due to the GEJE differed between the physically damaged areas and those affected by radiation. We also found the difference in the trend by qualifications: the reduction in total nursing staff per population was larger in Sousou, the area most affected by radiation, than in any other SMAs. Moreover, the number of nursing aides was most affected among the three types of staff. To promote the post-GEJE reconstruction of medical care systems, it might be necessary to develop policies to secure both nurses and nursing aides after nuclear disasters.

## Background

The Great East Japan Earthquake (GEJE) struck on 11 March 2011. The magnitude of the earthquake was 9.0 on the Richter scale, which was the fourth largest worldwide since 1990. Following the earthquake and subsequent tsunamis, there was an explosion at the Fukushima Daiichi Nuclear Power Plant (NPP). By 10 May 2014, 15 883 people were dead, 2676 were missing, and 130 000 houses were completely destroyed [1]. Health care facilities in the hardest hit areas were also severely damaged [2-4].

These areas had faced a shortage of health care resources for a long time, but the further depletion of health care workers due to the disaster is a growing concern [5]. Health care providers are also more willing to work in natural disaster areas than in radiological or nuclear disaster areas [6]. Particularly in Fukushima, the shortage of nurses is concerning because not only did an earthquake and tsunami cause damage but so too did a nuclear disaster [7].

Reconstruction of the health care system after the GEJE is proceeding; however, progress is reported to be slow and uneven across damaged areas [1]. We assumed that there might be a different trend in health care resources before and after the GEJE by the difference in regional damages. However, as far as we know, no study has examined the temporal trends of the nursing workforce – such as nurses, associate nurses and nursing aides – in the GEJE-afflicted areas. Nurses comprise most of the health care providers and work on the frontline and contribute to patient outcome [8]; therefore, securing nursing staff is an important issue in health care policy [9]. Although nurse and midwife personnel per thousand population of Japan was 11.489 and the 12th highest in the world [10], the maldistribution and relative shortage of nurses in rural areas in Japan has been of concern [11].

We aimed to describe the temporal trends in the geographic distribution of nursing staff before and after the GEJE, both in total and by job role, per population in the secondary medical areas (SMAs) of Iwate, Miyagi and Fukushima prefectures, which were most affected by the GEJE.

## Methods

### Study design

We conducted a longitudinal study at four time points (July 2007, 2010, 2011 and 2013) before and after the GEJE.

### Study setting

#### Study area

**Figure 1:**
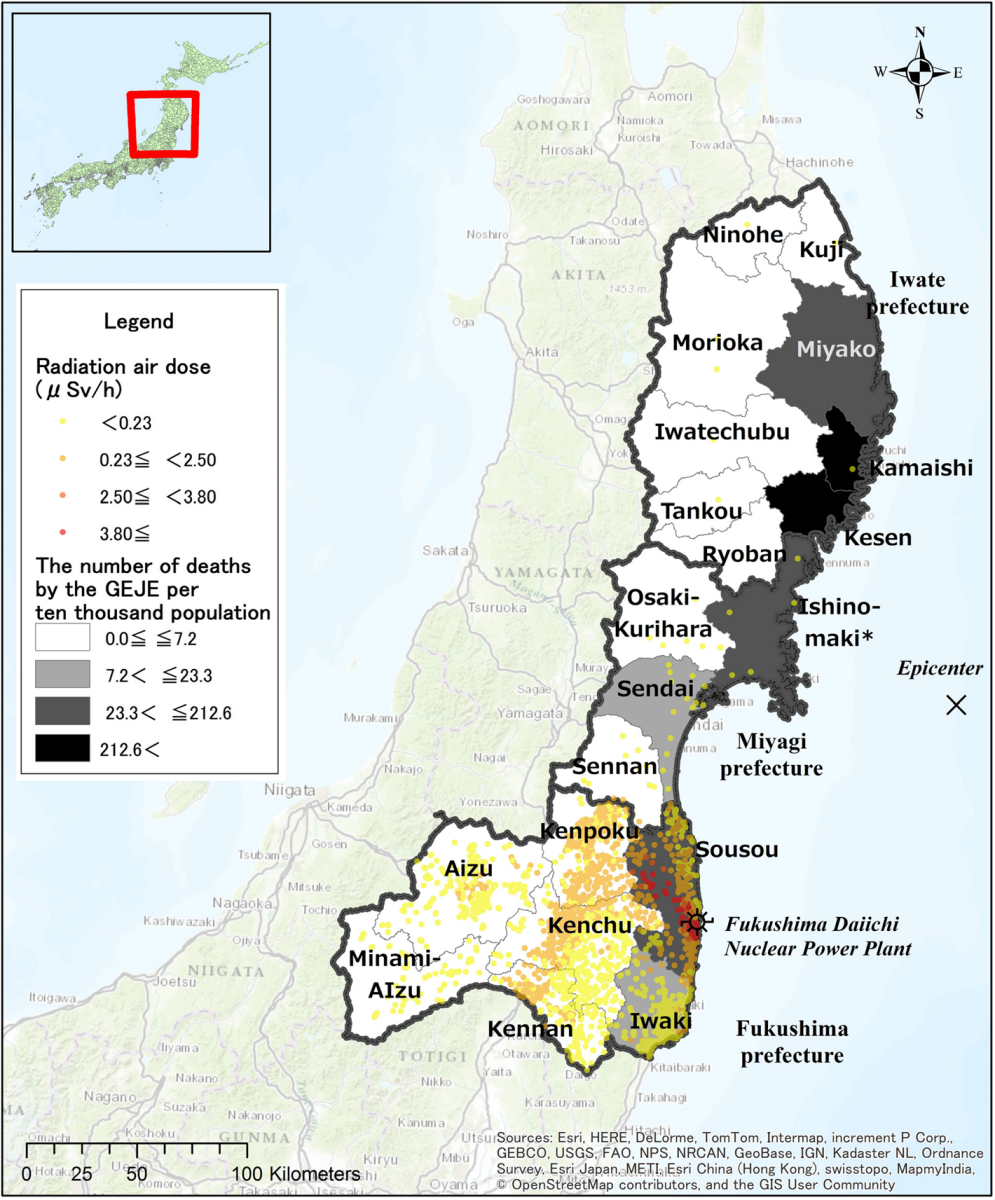
Damage caused by the Great East Japan Earthquake (GEJE) in Iwate, Miyagi and Fukushima prefectures.

*The GEJE-afflicted areas in Iwate, Miyagi and Fukushima prefectures were our study areas* (Figure 1)*.* An SMA is a geographical zone for operation of hospital, admission and emergency services. SMA boundaries are defined and revised by each prefecture under the Medical Care Act [12]. Most SMAs are based on a complex of adjacent municipalities, and there are several SMAs in a prefecture. We used the categories of SMAs defined by each prefectural hospital and health planning in 2013 [13-15].

#### Qualification of total nursing staff in Japan

In Japan, nurse and associate nurse qualifications are prescribed by the Act on Public Health Nurses, Midwives, and Nurses [16]. Nurses require a national licence, but associate nurses are qualified by prefectural governors, and both licensures are effective anywhere in Japan. Nurses have to study for at least 3 years at nursing school or university, whereas associate nurses have to study for only 2 years. Although the practical training and educational achievements differ between the two types of nurses, associate nurses are permitted to provide nursing services under the direction of a physician, dentist or nurse. Nursing aides are not required to be qualified and assist nurses in providing personal care of patients under nurses’ supervision and housekeeping work, such as washing laundry, cleaning up and clerical tasks in hospitals [17].

### Variables and data sources

#### Damage caused by the GEJE

Each prefectural government identified the number of deaths and collapsed houses caused by the GEJE as the indicators of damage [18-20]. We depicted the mortality in each SMA, which was calculated by the number of deaths per 10 000 population in 2010 [21-23], as an indicator of the physical damage caused by the GEJE (Figure 1). As Garber et al. [24] identified the heavily affected areas by disaster by flooded or having extensive or catastrophic damage, we referred to the high mortality area as the physically damaged area. According to the UNISDR, loss of life, or mortality, is one of the key indicators of disaster impacts in general [25]. As for the effect of the NPP accident, we pointed the atmospheric radiation dose rate (μSv/h) as on 19 November 2013, which was the latest available data during the study period (Figure 1), using the categories of the Japanese government. The Japanese government set the cut-off points for the atmospheric radiation dose rate as follows: (1) 0.23 μSv/h, which is equivalent to 1 mSv/year under the assumption of a life pattern of 16 h indoor (wooden construction) and 8-h outdoor activity per day, and for higher rates, it is necessary for national governments to carry out measures for decontamination; (2) 2.5 μSv/h, which is equivalent to 5 mSv/year under the assumption of 40 h/week working outdoors, and for higher rates, it is necessary for the government to measure workers’ exposure dose; and (3) 3.8 μSv/h, which is equivalent to 20 mSv/year under the assumption of a life pattern of 16 h indoor (wooden construction) and 8-h outdoor activity per day, and the government designates the area as an evaluation area [26,27]. We obtained the atmospheric radiation dose rate from establishment of the Extension Site of Distribution Map of Radiation Dose [28].

#### Number of total nursing staff

The numbers of nurses, associate nurses and nursing aides were obtained from full-time equivalent (FTE) hospital employees. The number of FTE hospital employees for part-time personnel was calculated by dividing the full-time worker’s working time by actual working time during June. Nurses included midwives and public health nurses who were employed by hospitals. The number of total nursing staff was the sum of the number of nurses, associate nurses and nursing aides.

The number of total nursing staff at each hospital in 2007, 2010, 2011 and 2013 was obtained from reports about basic hospitalization charges [29]. We collected these reports from all hospitals in Iwate, Miyagi and Fukushima prefectures by requesting disclosure of administrative documents from the Tohoku Regional Bureau of Health and Welfare on 15 October 2013. All hospitals have to submit their routine reports for the reimbursement of public health insurance based on actual data during June to each Regional Bureau of Health and Welfare office in July every year. These reports included the following information: hospital address (municipality); ownership; type of wards (for example, general, long-term care and psychiatric); number of beds; numbers of nurses, associate nurses and nursing aides; and daily ratio of total nursing staff per inpatients. Nationwide data for reference were collected by hospital reports, which are submitted on 1 October annually [30].

The population of the municipalities in each prefecture was obtained from the estimated population on 1 October annually [21-23]. The nationwide population for reference was acquired from the estimated population on 1 October annually [31].

#### Geographical data

Data on the boundaries of municipalities were obtained from the National Land Numerical Information Service provided by the Ministry of Land, Infrastructure, Transport, and Tourism [32]. We created the SMA boundaries using the above data. In this study, we used ArcGIS version 10.1 (ESRI Inc., Redlands, CA, USA) for the geographical analysis.

### Statistical analysis

Descriptive statistics about total nursing staff and population were collected by SMAs. We also calculated the ratio of total nursing staff per 1000 in the total population and as separated by qualifications in all SMAs from July 2010 to July 2013, to show the changes in the ratio before and after the GEJE. All analyses were performed with Stata version 13.1 (Stata Corp., College Station, TX, USA).

## Results

Table 1 shows the temporal trends of the total number of population, hospitals and total nursing staff in Japan. The ratio of total nursing staff per population shows a steady increase from 2007 to 2013.

**Table 1:** **Trend characteristics before and after the Great East Japan Earthquake (GEJE) in Japan**

**Variables**	**Before GEJE**		**After GEJE**	
	**2007**	**2010**	**2011**	**2013**
Population (thousand persons)	127 771	128 057	127 799	127 298
Number of hospitals	8 876	8 685	8 620	8 553
Nursing staffs per thousand	7.87	8.31	8.45	8.77
Nurses per thousand	4.99	5.52	5.71	6.08
Associate nurses per thousand	1.38	1.26	1.20	1.12
Nursing aides per thousand	1.49	1.53	1.54	1.57

Note: Population at 1 October in each year.

Number of hospitals and nursing staff on 1 October in each year.

### Location and damage from the GEJE in SMAs

There are 20 SMAs (9 in Iwate, 4 in Miyagi and 7 in Fukushima) within the study areas. In coastal SMAs, the mortality caused by the GEJE was higher than those in inland SMAs (Figure 1).

### SMA characteristics

Table 2 shows the temporal trends of the population, hospitals and total nursing staff in the SMAs in Iwate, Miyagi and Fukushima prefectures. The total number of hospitals in the three prefectures as of July 2007, 2010, 2011 and 2013 were 389, 380, 348 and 359, respectively. The maximum population in 2013 was 1.5 million in Sendai, Miyagi, and the minimum was 28 282 in Minamiaizu, Fukushima. In all SMAs, excluding Sendai and Ryouban, the population tended to decrease from 2007 to 2013. In coastal SMAs (Kamaishi, Kesen, Ishinomaki–Tome–Kesennuma (Ishinomaki), Sennan, Sousou and Iwaki), the number of hospitals and total nursing staff tended to decrease from 2010 to 2011.

**Table 2:** Secondary Medical Area (SMA) characteristics of Iwate, Miyagi, and Fukushima prefectures

Prefecture	Secondary Medical Area (SMA)	Variables	Before GEJE		After GEJE	
			2007 (changes in % from 2010)^b^	2010 (reference)	2011 (changes in % from 2010)^b,c^	2013 (changes in % from 2010)^b,c^
Iwate Prefecture	Kuji	Population	64 843 (3.7)	62 505	*61 535 (−1.6)*	*60 142 (−3.8)*
		Number of hospitals	4 (0.0)	4	4 (0.0)	4 (0.0)
		Number of nursing staff	408 (−5.3)	431	*413 (−4.2)*	*418 (−3.0)*
	Ninohe	Population	63 169 (4.2)	60 605	*59 708 (−2.5)*	*57 913 (−4.4)*
		Number of hospitals	3 (0.0)	3	3 (0.0)	3 (0.0)
		Number of nursing staff	420 (−6.0)	447	458 (2.5)	454 (1.6)
	Miyako	Population	93 529 (0.9)	92 694	*89 176 (−3.8)*	*86 302 (−6.9)*
		Number of hospitals	7 (16.7)	6	*5 (−16.7)*	*5 (−16.7)*
		Number of nursing staff	706 (−6.5)	755	756 (0.1)	*708 (−6.2)*
	Morioka	Population	486 107 (0.9)	481 699	482 096 (0.1)	*481 217 (−0.1)*
		Number of hospitals	42 (2.4)	41	*39 (−4.9)*	*39 (−4.9)*
		Number of nursing staff	4735 (−5.2)	4994	5024 (0.6)	5451 (9.2)
	Kamaishi	Population	57 496 (4.8)	54 850	*49 952(−8.9)*	*48 153(−12.2)*
		Number of hospitals	6 (0.0)	6	*5 (−16.7)*	*5 (−16.7)*
		Number of nursing staff	560 (−1.2)	567	*505 (−10.9)*	*548 (−3.4)*
	Iwatechubu	Population	236 254 (2.5)	230 509	*229 713 (−0.3)*	*227 303 (−1.4)*
		Number of hospitals	14 (7.7)	13	13 (0.0)	13 (0.0)
		Number of nursing staff	1331 (−5.9)	1415	1490 (5.3)	1448 (2.3)
	Kesen	Population	72 755 (3.6)	70 227	*65 552 (−6.7)*	*64 169 (−8.6)*
		Number of hospitals	4 (33.3)	3	*2 (−33.3)*	3 (0.0)
		Number of nursing staff	388 (−5.6)	411	*388 (−5.6)*	420 (2.2)
	Tankou	Population	144 795 (2.6)	141 071	*140 066 (−0.7)*	*137 659 (−2.4)*
		Number of hospitals	10 (0.0)	10	10 (0.0)	10 (0.0)
		Number of nursing staff	973 (0.2)	971	1013 (4.3)	997 (2.7)
	Ryouban	Population	131 991 (4.0)	126 923	134 958 (6.3)	131 595 (3.7)
		Number of hospitals	10 (0.0)	10	*9 (−10.0)*	*9 (−10.0)*
		Number of nursing staff	1017 (0.5)	1012	1047 (3.5)	*1007 (−0.5)*
Miyagi Prefecture	Ishinomaki^a^	Population	398 991 (2.7)	388 667	*367 725 (−5.4)*	*358 816 (−7.7)*
		Number of hospitals	25 (0.0)	25	*16 (−36.0)*	*20 (−20.0)*
		Number of nursing staff	2465 (−3.8)	2563	*2065 (−19.4)*	*2321 (−9.4)*
	Osaki-Kurihara	Population	293 307 (2.7)	285 721	*284 387 (−0.5)*	*279 962 (−2.0)*
		Number of hospitals	27 (0.0)	27	27 (0.0)	*26 (−3.7)*
		Number of nursing staff	1935 (0.5)	1926	2000 (3.8)	2057 (6.8)
	Sendai	Population	1 468 320 (−1.5)	1 490 098	*1 488 608 (−0.1)*	1 509 985 (1.3)
		Number of hospitals	77 (−3.8)	80	*78 (−2.5)*	81 (1.3)
		Number of nursing staff	10 135 (−9.9)	11 243	11 407 (1.5)	12 058 (7.2)
	Sennan	Population	188 381 (2.6)	183 679	*182 504 (−0.6)*	*179 380 (−2.3)*
		Number of hospitals	13 (0.0)	13	*12 (−7.7)*	13 (0.0)
		Number of nursing staff	1080 (0.7)	1072	*1049 (−2.1)*	1196 (11.6)
Fukushima Prefecture	Sousou	Population	198 390 (1.2)	195 950	*185 575 (−5.3)*	*180 194 (−8.0)*
		Number of hospitals	17 (6.3)	16	*6 (−62.5)*	*9 (−43.8)*
		Number of nursing staff	1485 (2.4)	1450	*467 (−67.8)*	*698 (−51.9)*
	Kenpoku	Population	505 531 (1.7)	497 059	*489 068 (−1.6)*	*478 602 (−3.7)*
		Number of hospitals	34 (6.3)	32	32 (0.0)	32 (0.0)
		Number of nursing staff	3587 (−10.7)	4018	4054 (0.9)	4017 (0.0)
	Aizu	Population	271 634 (3.7)	262 051	*259 617 (−0.9)*	*253 301 (−3.3)*
		Number of hospitals	19 (0.0)	19	19 (0.0)	*18 (−5.3)*
		Number of nursing staff	2725 (−3.8)	2833	2953 (4.2)	*2812 (−0.7)*
	Kenchuu	Population	558 249 (1.2)	551 745	*542 444 (−1.7)*	*533 286 (−3.3)*
		Number of hospitals	34 (3.0)	33	*31 (−6.1)*	33 (0.0)
		Number of nursing staff	4571 (−1.9)	4660	*4646 (−0.3)*	4864 (4.4)
	Iwaki	Population	350 258 (2.3)	342 249	*334 280 (−2.3)*	*327 856 (−4.2)*
		Number of hospitals	29 (0.0)	29	*26 (−10.3)*	*26 (−10.3)*
		Number of nursing staff	2973 (−4.5)	3112	*2926 (−6.0)*	*2923 (−6.1)*
	Kennan	Population	152 517 (1.6)	150 117	*148 595 (−1.0)*	*146 059 (−2.7)*
		Number of hospitals	13 (44.4)	9	10 (11.1)	9 (0.0)
		Number of nursing staff	1097 (11.0)	988	1111 (12.4)	1085 (9.8)
	Minamiaizu	Population	31 773 (6.3)	29 893	*29 416 (−1.6)*	*28 282 (−5.4)*
		Number of hospitals	1 (0.0)	1	1 (0.0)	1 (0.0)
		Number of nursing staff	88 (−3.3)	91	101 (11.0)	94 (3.3)
Total		Population	5 768 290 (1.2)	5 698 312	*5 624 975 (−1.3)*	*5 570 176 (−2.2)*
		Number of hospitals	389 (2.4)	380	*348 (−8.4)*	*359 (−5.5)*
		Number of nursing staff	42 679 (−5.1)	44 959	*43 873(−2.4)*	*45 576 (1.4)*

^a^Ishinomaki–Tome–Kesennuma.

^b^Changes in % in reference to 2010.

^c^Figures after the Great East Japan Earthquake. Negative change is shown in italic text.

### Trends in the ratio of total nursing staff per population

Table 3 shows the temporal trends of the ratio of total nursing staff and by qualification per population in Iwate, Miyagi and Fukushima prefectures. The maximum ratio of total nursing staff per 1000 population in 2013 was 11.38 in Kamaishi, and the minimum was 3.32 in Minamiaizu. In most SMAs, the ratio of total nursing staff per population tended to increase from 2007 to 2013. However, in Kuji, Kamaishi, Ryouban, Ishinomaki, Sennan, Sousou and Iwaki, the ratio of total nursing staff per population decreased from 2010 to 2011.

**Table 3:** Trends in nursing staff per thousand population by qualifications in Iwate, Miyagi and Fukushima prefectures

Prefecture	SMA	Variables	Before GEJE		After GEJE	
			2007 (changes in % from 2010)^b^	2010 (reference)	2011 (changes in % from 2010)^b,c^	2013 (changes in % from 2010)^b,c^
Iwate Prefecture	Kuji	Nursing staff	6.29 (−8.7)	6.90	*6.71 (−2.7)*	6.95 (0.8)
		Nurses	4.50 (−7.4)	4.86	*4.81 (−1.1)*	4.97 (2.2)
		Associate nurses	0.94 (8.9)	0.86	*0.86 (−0.3)*	*0.71 (−17.2)*
		Nursing aides	0.85 (−27.4)	1.17	*1.04 (−10.9)*	1.26 (8.2)
	Ninohe	Nursing staff	6.65 (−9.9)	7.38	7.67 (4.0)	7.84 (6.3)
		Nurses	5.13 (−11.4)	5.79	5.88 (1.5)	6.04 (4.4)
		Associate nurses	0.82 (24.7)	0.66	*0.60 (−8.6)*	*0.54 (−18.9)*
		Nursing aides	0.70 (−24.6)	0.92	1.19 (28.7)	1.26 (36.4)
	Miyako	Nursing staff	7.55 (−7.3)	8.15	8.48 (4.1)	8.20 (0.7)
		Nurses	4.83 (−6.9)	5.19	5.29 (2.0)	5.21 (0.5)
		Associate nurses	1.32 (24.4)	1.06	1.09 (2.9)	*1.05 (−0.3)*
		Nursing aides	1.40 (−26.2)	1.90	2.10 (10.4)	1.94 (1.9)
	Morioka	Nursing staff	9.74 (−6.0)	10.37	10.42 (0.5)	11.33 (9.3)
		Nurses	7.13 (−7.9)	7.75	7.84 (1.2)	8.47 (9.4)
		Associate nurses	1.13 (9.5)	1.03	*1.00 (−3.5)*	*0.96 (−6.7)*
		Nursing aides	1.48 (−6.9)	1.59	*1.58 (−0.3)*	1.89 (19.0)
	Kamaishi	Nursing staff	9.74 (−5.8)	10.34	*10.11 (−2.2)*	11.38 (10.1)
		Nurses	7.08 (−4.4)	7.40	*7.37 (−0.5)*	8.16 (10.3)
		Associate nurses	1.39 (−2.2)	1.42	*1.34 (−5.7)*	*1.25 (−12.4)*
		Nursing aides	1.27 (−16.1)	1.51	*1.40 (−7.4)*	1.97 (30.4)
	Iwatechubu	Nursing staff	5.63 (−8.2)	6.14	6.49 (5.7)	6.37 (3.8)
		Nurses	4.43 (−5.5)	4.69	4.90 (4.5)	4.73 (0.9)
		Associate nurses	0.63 (13.5)	0.56	0.57 (2.7)	*0.50 (−11.2)*
		Nursing aides	0.57 (−36.2)	0.89	1.01 (13.6)	1.14 (28.1)
	Kesen	Nursing staff	5.33 (−8.9)	5.85	5.92 (1.1)	6.55 (11.8)
		Nurses	4.40 (3.0)	4.27	4.36 (2.1)	4.96 (16.0)
		Associate nurses	0.56 (−20.8)	0.71	*0.70 (−1.4)*	*0.56 (−21.2)*
		Nursing aides	0.37 (−57.3)	0.87	*0.85 (−1.6)*	1.03 (18.4)
	Tankou	Nursing staff	6.72 (−2.4)	6.88	7.23 (5.1)	7.24 (5.2)
		Nurses	4.68 (−4.1)	4.88	5.20 (6.6)	*4.84 (−0.8)*
		Associate nurses	0.90 (12.0)	0.81	*0.81 (−0.2)*	0.84 (3.4)
		Nursing aides	1.14 (−4.0)	1.20	1.23 (2.5)	1.57 (31.0)
	Ryouban	Nursing staff	7.71 (−3.4)	7.97	*7.76 (−2.7)*	*7.65 (−4.0)*
		Nurses	5.95 (−4.9)	6.26	*5.98 (−4.4)*	*6.00 (−4.0)*
		Associate nurses	0.94 (21.7)	0.77	*0.76 (−1.2)*	*0.62 (−20.3)*
		Nursing aides	0.82 (−13.5)	0.95	1.02 (7.4)	1.03 (9.3)
Miyagi Prefecture	Ishinomakia	Nursing staff	6.18 (−6.3)	6.59	*5.62 (−14.8)*	*6.47 (−1.9)*
		Nurses	3.90 (−8.5)	4.26	*3.79 (−11.0)*	4.36 (2.2)
		Associate nurses	1.28 (4.6)	1.23	*0.94 (−23.1)*	*0.94 (−23.2)*
		Nursing aides	0.99 (−10.1)	1.10	*0.88 (−20.4)*	1.17 (5.8)
	Osaki-Kurihara	Nursing staff	6.60 (−2.1)	6.74	7.03 (4.3)	7.35 (9.0)
		Nurses	3.68 (−9.0)	4.04	4.20 (4.0)	4.57 (13.1)
		Associate nurses	1.69 (21.6)	1.39	1.44 (3.5)	*1.35 (−3.3)*
		Nursing aides	1.23 (−6.2)	1.31	1.39 (6.1)	1.43 (9.4)
	Sendai	Nursing staff	6.90 (−8.5)	7.55	7.66 (1.6)	7.99 (5.8)
		Nurses	5.12 (−10.3)	5.70	5.82 (2.1)	6.15 (7.8)
		Associate nurses	0.89 (9.8)	0.81	*0.73 (−10.2)*	*0.65 (−19.8)*
		Nursing aides	0.90 (−13.3)	1.03	1.12 (7.9)	1.19 (15.0)
	Sennan	Nursing staff	5.73 (−1.8)	5.84	*5.75 (−1.5)*	6.67 (14.2)
		Nurses	2.84 (−7.2)	3.06	3.15 (2.8)	3.70 (20.8)
		Associate nurses	1.57 (15.9)	1.36	*1.14 (−15.9)*	*1.18 (−13.2)*
		Nursing aides	1.32 (−7.0)	1.42	1.46 (3.0)	1.80 (26.3)
Fukushima Prefecture	Sousou	Nursing staff	7.49 (1.2)	7.40	*2.52 (−66.0)*	*3.87 (−47.7)*
		Nurses	3.28 (−6.0)	3.49	*1.70 (−51.2)*	*2.31 (−33.7)*
		Associate nurses	2.56 (17.0)	2.19	*0.45 (−79.6)*	*0.93 (−57.7)*
		Nursing aides	1.64 (−4.5)	1.72	*0.37 (−78.7)*	*0.63 (−63.2)*
	Kenpoku	Nursing staff	7.10 (−12.2)	8.08	8.29 (2.5)	8.39 (3.8)
		Nurses	4.37 (−16.1)	5.21	5.45 (4.6)	5.67 (9.0)
		Associate nurses	1.59 (9.5)	1.46	*1.36 (−6.4)*	*1.23 (−15.8)*
		Nursing aides	1.13 (−20.4)	1.42	1.48 (4.2)	1.49 (5.2)
	Aizu	Nursing staff	10.03 (−7.2)	10.81	11.37 (5.2)	11.10 (2.7)
		Nurses	6.27 (−6.9)	6.73	7.20 (6.9)	7.18 (6.7)
		Associate nurses	2.21 (−4.8)	2.32	2.33 (0.4)	*2.12 (−8.9)*
		Nursing aides	1.55 (−11.7)	1.76	1.85 (5.1)	1.80 (2.8)
	Kenchuu	Nursing staff	8.19 (−3.1)	8.45	8.56 (1.4)	9.12 (8.0)
		Nurses	4.97 (−8.1)	5.40	5.68 (5.1)	6.19 (14.5)
		Associate nurses	1.69 (9.8)	1.54	*1.40 (−8.6)*	*1.31 (−14.6)*
		Nursing aides	1.54 (1.9)	1.51	*1.48 (−1.5)*	1.62 (7.6)
	Iwaki	Nursing staff	8.49 (−6.7)	9.09	*8.75 (−3.7)*	*8.92 (−1.9)*
		Nurses	4.64 (−10.6)	5.19	*5.06 (−2.5)*	5.29 (1.9)
		Associate nurses	2.31 (8.6)	2.12	*1.87 (−12.1)*	*1.78 (−16.0)*
		Nursing aides	1.54 (−13.4)	1.78	1.82 (2.7)	1.84 (3.7)
	Kennan	Nursing staff	7.19 (9.3)	6.58	7.48 (13.6)	7.43 (12.9)
		Nurses	3.86 (−9.9)	4.28	4.72 (10.5)	5.11 (19.4)
		Associate nurses	2.29 (57.3)	1.46	1.76 (20.9)	*1.43 (−1.9)*
		Nursing aides	1.04 (23.2)	0.85	0.99 (16.9)	0.89 (5.2)
	Minamiaizu	Nursing staff	2.77 (−9.0)	3.04	3.43 (12.8)	3.32 (9.2)
		Nurses	2.39 (−14.9)	2.81	2.99 (6.5)	2.90 (3.2)
		Associate nurses	0.25 (50.5)	0.17	0.27 (62.6)	0.25 (48.0)
		Nursing aides	0.13 (88.2)	0.07	0.17 (154.1)	0.18 (164.2)
Total		Nursing staff	7.40 (−6.2)	7.89	*7.80 (−1.1)*	8.18 (3.7)
		Nurses	4.87 (−9.1)	5.36	*5.41 (−1.1)*	5.75 (7.3)
		Associate nurses	1.38 (−11.3)	1.24	*1.10 (−11.1)*	*1.03 (−16.8)*
		Nursing aides	1.15 (−11.0)	1.30	*1.29 (−0.7)*	1.40 (8.4)

^a^Ishinomaki–Tome–Kesennuma.

^b^Changes in % in reference to 2010.

^c^Figures after the Great East Japan Earthquake. Negative change is shown in italic text.

According to qualification, the ratio of nurses per population tended to increase from 2007 to 2013 in 14 SMAs, excluding Kuji, Kamaishi, Ryouban, Ishinomaki, Sousou and Iwaki. In these six SMAs, the ratio of nurses tended to increase from 2007 to 2010, decrease from 2010 to 2011 and then increase again after 2011. The ratio of associate nurses per population peaked in 2007 and has decreased since then in most SMAs. The ratio of nursing aides per population tended to increase from 2007 to 2013 in 14 SMAs, excluding Kuji, Kamaishi, Kesen, Ishinomaki, Sousou and Iwaki. In these six SMAs, the ratio of nursing aides decreased from 2010 to 2011 and increased again after 2011. In Sousou, the reductions in the ratio for all qualifications from 2010 to 2011 were the largest compared with other SMAs. The percentages of nurses, associate nurses and nursing aides were −51.2%, −79.6% and −78.7%, respectively.

### Changes before and after the GEJE

**Figure 2:**
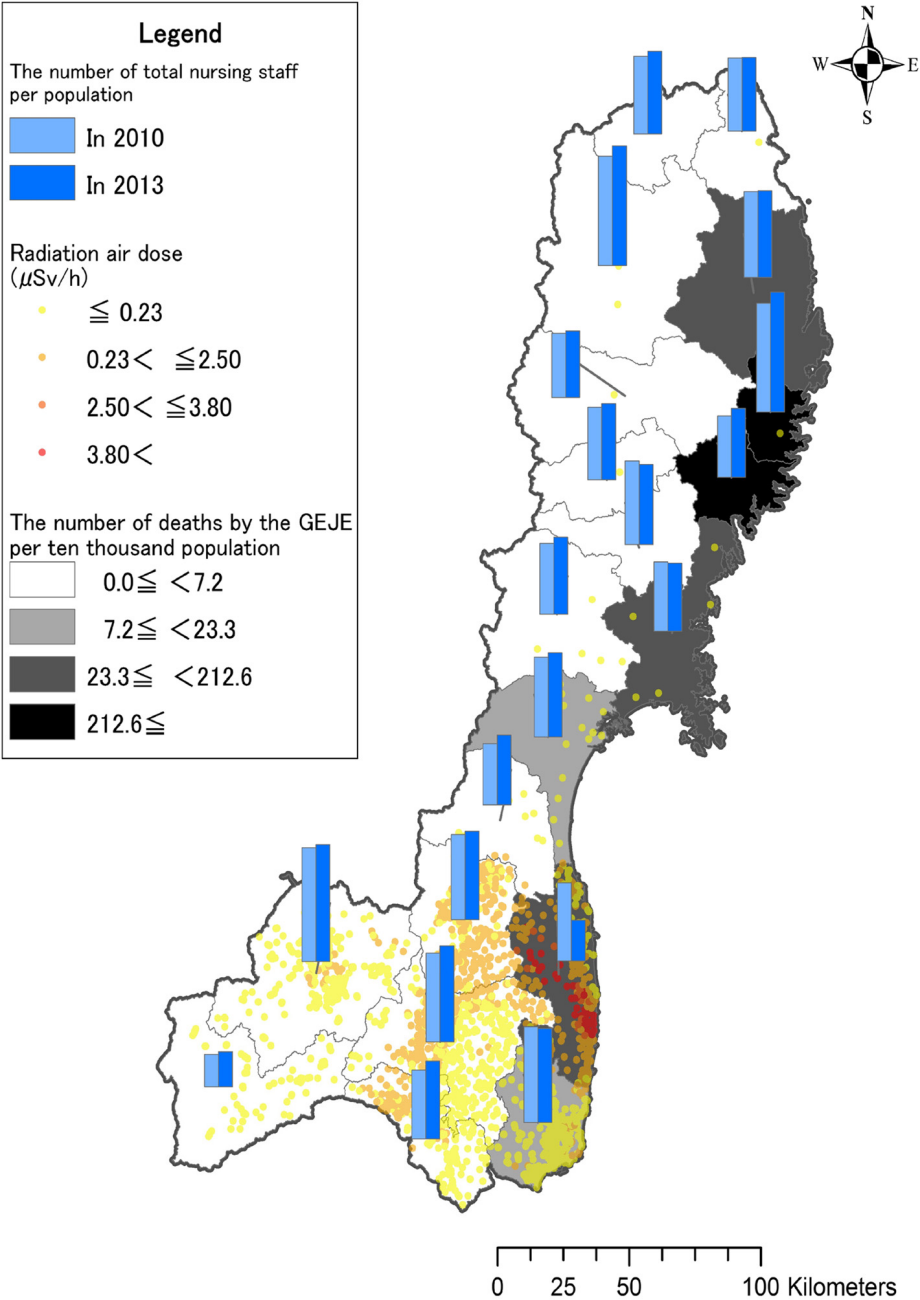
Number of total nursing staff per population in 2010 and in 2013.

**Figure 3:**
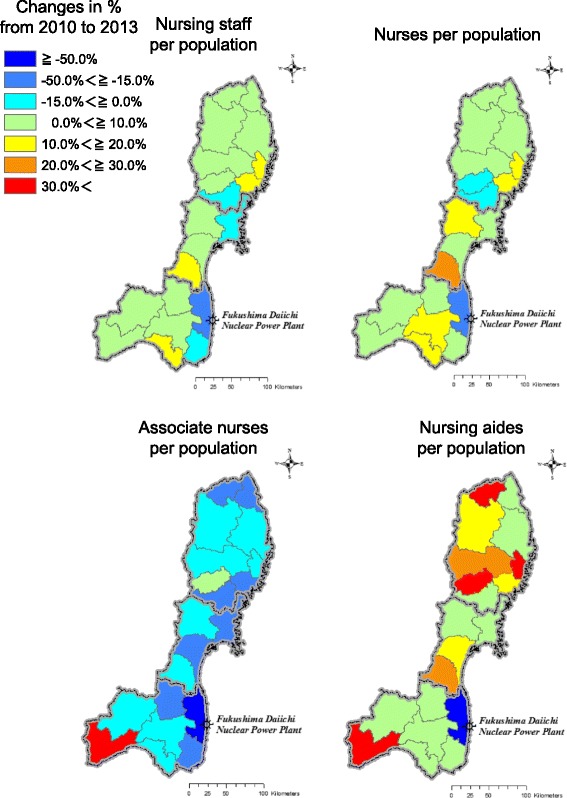
Changes in the ratio of nursing staff per population by qualification from 2010 to 2013.

Changes in the ratio of total nursing staff and by qualification per population from 2010 to 2013 are shown in Table 3, Figures 2 and 3. The percent changes in total nursing staff per population were negative in four SMAs: Ryouban (−4.0%), Ishinomaki (−1.9%), Sousou (−47.7%) and Iwaki (−1.9%). According to qualifications, the percent changes in nurses per population were negative in three SMAs: Tankou (−0.8%), Ryouban (−4.0%) and Sousou (−33.7%). Those of associate nurses were negative in all SMAs excluding Tankou (3.4%) and Minamiaizu (48.0%), and the largest decrease was found in Sousou (−57.7%). A large decrease in the ratio of nursing aides was found in Sousou (−63.2%), while the ratios of nursing aides increased in all the other SMAs. For all qualifications, the ratio of total nursing staff per population in Sousou decreased the most.

## Discussion

Our study indicated that the trend in the ratio of total nursing staff per population after the GEJE was different between the physically damaged areas and those affected by radiation. In coastal SMAs, which were greatly damaged by tsunamis, the ratio of total nursing staff per population greatly decreased immediately after the GEJE, as indicated previously [2-5]. In most SMAs, because the reconstruction of hospitals was progressing after the GEJE, the ratio of total nursing staff per population increased and exceeded the pre-GEJE level for 2 years. In four SMAs (Ryouban, Ishinomaki, Sousou and Iwaki), the ratio of total nursing staff per population had not yet recovered to the pre-GEJE level. In Ryouban, Ishinomaki and Iwaki, the ratio of total nursing staff per population achieved almost 96% and over before the GEJE. In the inland area of Ryouban, the decrease in total nursing staff per population was caused by the post-GEJE increase in population. In Ishinomaki and Iwaki, which were one of the greatest damaged by the tsunami, the decrease in total nursing staff per population was explained by the decrease in associate nurses.

In Sousou, the SMA where the NPP is located and which was also severely damaged by the earthquake and tsunamis, the total nursing staff per population in 2013 was only half of that in 2010. The number of physicians per 100 000 population also decreased by 34.4% (from 120.4 on 31 December 2010 to 79.0 on 31 December 2012) in Sousou [33], which was almost the same as the reduction of nurses per population. These large and continuous decreases of nursing staffs might be explained by the following reasons. First, the decrease in the number of hospitals might cause a decrease in the ratio of total nursing staff per population. After the GEJE, the Japanese government designated three categories of areas to which evacuation orders were issued to prevent further expansion of the damage caused by the NPP accident: Area 1, where it is confirmed that the annual cumulative dose will definitely be ≤20 mSv, is an area in which evacuation orders have already been lifted; Area 2, where the annual cumulative dose from the present moment is expected to be >20 mSv and where residents are ordered to remain evacuated in order to reduce the risk of radiation exposure; and Area 3, where the annual cumulative dose is expected be ≥20 mSv for a long time, especially within 5 years, and the current annual cumulative dose is >50 mSv, and where it is expected that residents will face difficulties in returning for a long time [34,35]. Most parts of Areas 1 and 2 have been included in Sousou SMA, and the hospitals located within the areas have been closed [4]. Health care workers who had worked in those hospitals had to move, even if they wanted to work there. Even in the hospitals that maintained their functions, the increased workload among the nursing staff, due to the initial significant reductions in the number of available staff after the GEJE, might encourage further departure of nursing staff [36] and discourage their return, which could result in a continuous shortage of nursing staff.

Another possible reason is the concern over radiation exposure among the nursing staff, which might have made them move to areas located far from the NPP. As previous studies have indicated, only half of the health care providers were willing to work in areas affected by nuclear disasters [6,37−40]. The most frequently cited reason for employees’ unwillingness to report for duty during a disaster was fear and concern for the safety of their families and themselves [37]. Although the surrounding SMAs have also suffered from high doses of radiation, the relatively higher radiation doses and the shorter distance from the NPP in Sousou might be a reason for the larger reduction and delayed return of the nursing staff. There might be some other factors that could be associated with the workforce distribution after the disaster. Although such factors would be strongly associated with the mortality or radiation doses in each SMA, further investigations are required to find those factors that are associated with the workforce distribution.

The change in the ratio of nursing staff per population differed according to qualifications, and the change in ratio of nursing aides was the lowest among the three types of nurses. Cone et al. indicated that willingness to work in a disaster area was higher for nurses than for nonclinical staff [39], but our findings indicated different tendencies. Because most nursing aides are irregular employees in Japan, they might be more likely to change their workplace than nurses are. Also, there might be differences in knowledge regarding radiation. Lanzonni et al. showed that physicians and nurses with knowledge regarding biological events had greater willingness to work in a biological disaster [40].

From the above findings, policy and actions to secure the different types of nursing staff are required, especially for the areas suffering from a continuous shortage of staff. The Fukushima prefectural government developed a policy for securing nurses after the GEJE: (1) establish a study fund aimed at the security of nurses and associate nurses, (2) support re-employment of latent nurses, (3) set aside a subsidy to assist with part of the personnel expenses in the hospitals forced to reduce operation by the GEJE to maintain employment of nurses and (4) strengthen the in-service training in the hospitals within Sousou [7,15]. These policies might be partly effective and contribute to the relatively higher ratios of nurses and associate nurses per population than nursing aides, even in Sousou SMA. To secure the overall nursing staff, however, more comprehensive approaches to enhance the return of nursing aides as well as nurses and associate nurses to the hospitals are required. As the skill mix of nursing staff has been promoted globally [41], a financial incentive was introduced to promote employment of nursing aides in hospitals, which was expected to reduce nurses’ workload and allow them to spend more time doing their own professional jobs such as bedside care [42]. According to a previous study, the motivation of nursing aides in Japan can be defined by the following factors: salary satisfaction, free time to do one’s own thing, nursing aides as important partners in the job, feeling helpful to patients, participating in decision-making and job-skill improvement [43]. Thus, policies including salary increase, strengthening in-service training and ensuring a pleasant working environment, might be effective in securing nursing aides in Sousou and, consequently, in providing a better work environment by reducing the overall workload of the nursing staff in each hospital. To prevent further reductions in staff and for future preparedness, education programmes regarding nuclear disasters could be effective, since such programmes were found to minimize health care providers’ fears and concerns about radiation [44].

In Minamiaizu, not only the ratio of total nursing staff per population but also the ratio of hospitals and physicians per population has been the lowest and much lower than the nationwide levels [45]. The relative shortage and misdistribution of health care providers in remote areas such as Minamiaizu has been a continuous policy concern in Japan [46-48].

To the best of our knowledge, the longitudinal impact of a disaster on the distribution of health care providers is unknown, especially during a nuclear accident. Therefore, this study is the first to show the longitudinal trend in the ratio of total nursing staff per population in SMAs according to qualifications before and after the GEJE.

There were some limitations to our study. First, the population might have been overestimated in those SMAs that were greatly damaged by the GEJE because of evacuation, without people changing area registration. Accordingly, the ratio of total nursing staff per population might have been underestimated in these areas. The Japanese government reported that there are about 133 309 people actually living around restricted areas and areas for which evacuation orders have been issued, such as in the Sousou SMA, excluding three municipalities (Shinchi Town, Soma City and Hirono Town), as of 31 December 2013 [35]. In our study, the population in the same area was 131 705 as of 1 October 2013. In considering these numbers, a perceived limitation of overestimation is not serious because the government’s calculation of Sousou’s population was almost the same as our estimation. Second, there were data limitations with regard to type of occupation, workplace and time. In our data, the data before 2007 and other health providers’ information did not exist. Trends in other health care providers, such as physicians, dentists and pharmacists, should be clarified in future studies. Total nursing staff included only hospital workers because of data availability. Trends in total nursing staff in other workplaces, such as clinics or long-term care facilities, should be investigated in the future. Third, our findings could not indicate the causal association between the numbers of total nursing staff and effects of radiation because we described the temporal trends using statistical data and did not assess the dynamic flows of nursing staff according to radiation dose. Therefore, further studies are required to investigate the causal association.

## Conclusions

We investigated the temporal trend in the total nursing staff per population before and after the GEJE. Our study indicated that the trend in total nursing staff per population ratio after the GEJE was different between the physically damaged areas and those affected by radiation. Nursing qualifications also showed differences in trends: the reduction in the ratio of total nursing staff per population was larger in Sousou – the area most affected by radiation – than in any other SMAs, and that of nursing aides was the largest compared with those of nurses and associate nurses. To promote the reconstruction of the medical care systems after the GEJE, policies for both nurses and nursing aides, considering the influences of radiation, might be required.

## Abbreviations

FTE: Full-time equivalent
GEJE: Great East Japan Earthquake
NPP: Nuclear power plant
SMA: Secondary medical area
